# Differential expression of cancer associated plasma miRNAs in the ordnance factory workers, exposed to 2,4,6-Trinitrotoluene

**DOI:** 10.12669/pjms.38.3.4928

**Published:** 2022

**Authors:** Muhammad Aamir, Abdul Khaliq Naveed, Muhammad Afzal, Amer Siddiq

**Affiliations:** 1 Muhammad Aamir, FCPS. Biochemistry Department, Riphah International University, Islamabad, Pakistan; 2 Abdul Khaliq Naveed, PhD. Biochemistry Department, Riphah International University, Islamabad, Pakistan; 3 Muhammad Afzal, PhD. Biochemistry Department, Riphah International University, Islamabad, Pakistan; 4 Amer Siddiq, FCPS. Biochemistry Department, Riphah International University, Islamabad, Pakistan

**Keywords:** TNT, miRNAs, RT-PCR

## Abstract

**Objectives:**

To determine differential expression of microRNAs (miRNAs) in plasma of 2, 4, 6-Trinitrotoluene (TNT) exposed ordnance factory workers.

**Methods:**

A case control study was conducted at the Department of Toxicology, Armed Forces Institute of Pathology, Rawalpindi from July to December 2020. A total 30 subjects were recruited from an ordnance factory that were directly exposed to TNT and 120 non-exposed individuals from non-factory healthy population. Plasma levels of five miRNAs including miRNA-let-7a-2, miRNA-34a-1, miRNA-21-2, miRNA-106b-1, miRNA-122a-1 were measured by quantitative real-time polymerase chain reaction (RT-PCR).

**Results:**

Micro RNAs showed a wide range of Ct (cycle threshold) values ranging from 23.48 to 41.94. Among the five miRNAs let-7a-2 and miRNA-122a-1 displayed relatively high expression with Ct values ranging from 26.58 ± 2.25 to 27.18 ± 0.80 respectively. Relative fold change expression for all five miRNAs of exposed individuals were found high (*p* <0.0001) vs non-exposed. Dividing fold change expression of exposed individuals into two groups as ≤ 10 and > 10, the individuals having ≤ 10-fold change expression were19 (63.3%) in miRNA-let-7a-2, 30 (100%) in miRNA-34a-1 and 23 (76.7%) in miRNA-122a-1 while in miRNA-21-2 and miR-106b-1, 23 (76.7%) and 18(60%) individuals had > 10-fold change expression respectively. Among the five miRNAs in exposed individuals, miRNA-let-7a-2, miR-21-2, miR-106b-1 and miR-122a-1 were found highly expressed with fold change expression > 10 (*p* <0.0001). No significant association was found between miRNAs expression levels with age and working duration.

**Conclusion:**

The study shows upregulation of all five miRNAs in TNT exposed subjects with no significant association of expression levels with age and working duration**.**

## INTRODUCTION

Two, 4, 6 Trinitrotoluene (TNT) is a yellow, odorless solid that has been commonly used as an explosive for military and industrial purposes since the beginning of the twentieth century^1^ and considered a serious environmental pollutant posing a grave risk to occupationally exposed population. The toxic effects of TNT were first noted during the First World War when many TNT workers were reported to have died from aplastic anemia or toxic hepatitis.^2^ Because of its toxic properties, TNT is considered as a possible human carcinogen, causing hepatocellular carcinoma, leukemias and bladder cancer.^3^

Considering association of TNT and its metabolites with different types of cancers, there is a need to identify markers for early detection of cancer in occupationally exposed persons to TNT. Recently, microRNAs (miRNAs) have come up as a promising diagnostic modality that discriminates different malignancies from their normal counterparts in early stages of cancer.^4^ They are short (19 to 25 nucleotides base-pairs), single stranded non-coding RNAs that regulate gene expression and cellular processes – differentiation, apoptosis and proliferation.^5^ Defective expression of some miRNAs that are associated with cancers, like hepatocellular carcinoma, leukemia and bladder cancer include miRNA-122, miRNA 21, miRNA let-7, miRNA-34a and miRNA -106.^6^-^10^

In the recent past, there has been renewed interest in elucidating biochemical, environmental and biological effects of TNT exposure on human health. Although TNT and various miRNAs have been associated with different cancers, yet no study is available which shows dysregulation of miRNAs in subjects chronically exposed to TNT. The present study was planned to find differential expression of cancer associated miRNAs among workers of Ordnance Factory, Wah City in Punjab province of Pakistan by comparison with non-exposed healthy controls.

## METHODS

### Study subjects and Sample preparation

The study protocol was approved by the Ethical Review Committee of the Basic Medical Sciences, Islamic International Medical College, Riphah International University, Pakistan (appl. # Riphah/IRC/21/39). The study subjects were recruited on the basis of non-probability convenient sampling method as exposed group (n = 30), working in Ordnance Factory who were directly exposed to TNT (manufacturing, handling, filling, and packaging) for more than two years and non-exposed individuals from non-factory healthy population (n=120). All the subjects included in the study had no history of any cancer, hepatitis B, hepatitis C and hepatic or hematological malignancy. A written informed consent was taken before drawing blood sample. Blood samples (3 ml each) were collected into EDTA tubes, centrifuged at 3000xg for 10 minutes at 2-8 ºC. Plasma was removed and transferred into RNase-free 2 ml microcentrifuge tubes and then kept at – 80 ºC in two aliquots until further processing for total RNA isolation.

### Total RNA isolation from plasma

Plasma samples were thawed at 2-8 ºC and then centrifuged at 16000xg for 5 minutes at 4 ºC to remove fat content and precipitates. The supernatant was then transferred into fresh 1.5 ml RNase-free tubes. A chemical-based method was used to extract total RNA by adding 250 ul of plasma into 750 ul trizol^LS^ (Invetrogen, ThermoFisher Scientific) and gently inverted 5 – 8 times and incubated for five minutes on freezing rack. After that, 200 ul chloroform was added. Samples were mixed by shaking and incubated for three minutes on ice rack. After which, samples were centrifuged at 12,000xg for 15 minutes, at 4ºC. The upper aqueous phase was then transferred into a fresh RNase-free-tubes into which 500 ul of 100 % isopropanol was added and further incubated on ice rack for 10 minutes after which centrifuged at 12000xg at 4ºC for 10 minutes. The supernatant was discarded without disturbing the pellet which was re-suspended by adding one ml of 100% ethanol, vortexed briefly, then centrifuged for five minutes at 7500xg at 4ºC and air dried for 15 minutes. The pellets were re-suspended in 20-50 µl of RNase-free water and incubated for 10 minutes at 55-60ºC. Purity and concentration of the total RNA was evaluated by NanoDropND-1000 UV-Vis Spectrophotometer (Thermo Scientific, Massachusetts, USA) at an optical density (OD) ratio of A260/230 nm and A260/280 nm.

### Reverse transcription (RT) and real-time polymerase chain reaction (RT-PCR)

Mature miRNAs were immediately converted into cDNA after polyadenylation with oligo-dT primer using miScript II RT Kit (Qiagen, Hilden, Germany). In a total volume of 20 ul reaction mixture, 200 ng of total RNA, 2 μl buffer (miScript nucleics mix), 2 μl miScript reverse transcriptase mix and 4 μl miScript HiFlex buffer were added and the volume was adjusted up to 20 μl with RNase free water. Reverse transcriptase reaction was run to convert miRNAs into cDNA for 60 minutes at 37°C and then for five minutes at 95°C in RT-PCR machine. Before setting-up PCR reaction, cDNA was diluted at a ratio of 1:5 with RNase-free water. Polymerase chain reaction was performed in a final volume of 25μl, comprising 2.5 μl diluted cDNA, 2.5 μl miScript universal primer, 12.5 μl SYBR green PCR master mix, and 2.5 μl assay specific forward primers for let-7a-2, miR-34a-1, miR-21-2, miR-106b-1, miR-122a-1 (Qiagen, Hilden, Germany). Threshold cycles (Ct) were measured by quantitative RT-PCR via use of miScript SYBR^®^ Green PCR kit (Qiagen, Hilden, Germany). Thermal cycling conditions for RT-PCR were as follows: initial denaturation at 95ºC for 15 minutes, followed by 45 cycles of 94ºC for 15 seconds, annealing for 30 seconds at 55ºC for miRNA-let-7a-2, miRNA-34a-1, miRNA-21-2, miRNA-122a-1, or 53 ºC for miRNA-106b-1 and extension at 70ºC for 30 seconds. Amplification was presented as cycle threshold (Ct) values, which indicates the number of cycles at which the fluorescent signal crosses the threshold line.

### Statistical Analysis

Absolute Ct values were represented as mean ± standard deviation (SD) of two or three independent experiments. A clustered boxplot was drawn to represent the dispersion of expression levels and the differences between the mean values of exposed and non-exposed groups for each miRNA were compared using the independent student t-test. Chi-square test was used to find out association between expression levels with age and working duration. Data were analyzed using SPSS version 22 (Software Inc, California, USA) and *p*-value of ≤ 0.05 was considered as statistically significant.

## RESULTS

A total of 30 cases working in Ordnance factory with ages ranging from 25–56 years (mean= 43 years) and working duration ranging from 4 – 34 (mean= 16 years) were included in the study. Five miRNAs were evaluated by assay specific real-time PCR assay after normalization with equivalence unit mass of total RNA extracted from equal volumes of plasma for each sample. One sample among the non-exposed samples was chosen as a calibrator and the expression of target miRNA in all other samples were taken as decreased or increased relative to that calibrator. The relative quantification of each miRNA was measured between exposed (n = 30) and non-exposed individuals (n = 120).

MicroRNAs showed a wide range of cycle threshold (Ct) values ranging from 23.48 to 41.94. A relatively high expression with mean Ct values ranging from 26.58 ± 2.25 to 27.18 ± 0.80 was displayed by miRNA-Let-7a-2 and miR-122a-1 respectively. Moderately expressed miRNAs were miR-21-2 and miR-34a-1 with mean Ct vales between 29.88 ± 1.63 to 30.69 ± 0.83 respectively and miR-106b-1 with least expression with mean Ct value 36.47 ± 1.75. Among five miRNAs, miR-34a-1 and miR-122a-1 showed the least variability. Mean and ranges of Ct values for all miRNAs are given in Table-I.

**Table-I:** Threshold cycle (Ct) values of miRNAs (in duplicate) in exposed individuals.

miRNAs	Range	Min	Max	Mean ± SD
Let-7a-2	9.66	23.48	33.14	26.58 ± 2.25
miR-34a-1	3.41	29.38	32.79	30.69 ± 0.83
miR-21-2	9.27	26.27	35.54	29.88 ± 1.63
miR-106b-1	7.39	34.55	41.94	36.47 ± 1.75
miR-122a-1	3.50	25.08	28.58	27.18 ± 0.80

**Fig.1:**
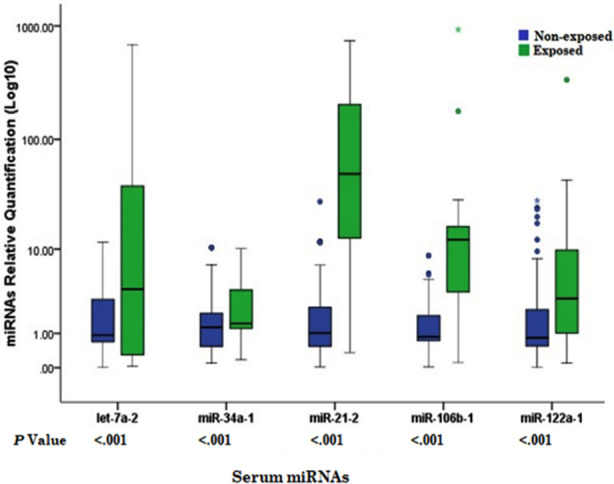
Differential expression of miRNAs in sera of exposed and non-exposed individuals.

Using Ct values, relative fold change expression of five miRNAs including miRNA-let-7a-2, miRNA-34a-1, miRNA-21-2, miRNA-106b-1, miRNA-122a-1 were further calculated by using the equation (test/calibrator) = 2^∆ Ct^). Logarithmical relative fold change expression for all five miRNAs of exposed individuals were found highly significant with *p* values <0.0001 compared with non-exposed group. Median and interquartile ranges with *p* values are indicated in Fig.1.

The relative fold change expression of miRNAs were determined by assay specific real-time PCR and normalised with equvalent unit mass of RNA input. Boxplot indicates median lines, interquartile ranges, error bars represent range of values and outliers (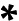). Relative expression of oncogenic and tumor supressor miRNAs of exposed (n=30) to non-exposed (n=120) was observed. Significant dysregulation of miRNAs indicated with *p* values; *p<0.001.

We further analyzed the fold change expression of exposed individuals into two groups taken as ≤ 10 and > 10 based on average variance of non-exposed individuals. Among cases having ≤ 10-fold change expression, 19 (63.3%) were observed in miRNA-let-7a-2, all thirty (100%) in miRNA 34a-1 and 23 (76.7%) in miRNA 122a-1, whereas 23 (76.7%) and 18 (60%) individuals had > 10 fold change expression in miRNAs 21-2 and 106b-1 respectively (Table-II). In mi RNAs let-7a-2, 21-2 and 122a-1, no statistically significant difference was observed in relative fold expression between exposed group having ≤ 10fold expression and nonexposed individuals (Table-III). However, a statistically significant difference was observed in relative fold expression of miRNA-34a-1 and miRNA-106b-1 between exposed group having ≤ 10fold expression and non-exposed individuals (Table-III). Among the five miRNAs in exposed individuals, let-7a-2, miR-21-2, miR 106b-1 and miR-122a-1 were found highly expressed with mean fold change expression > 10 (*p* <0.0001) as shown in (Table-III).

**Table-II:** Proportion of exposed individuals with fold change levels of miRNAs (N = 30).

miRNAs	Expression ≤ 10 n (%)	Expression > 10 n (%)	Total N (%)
Let-7a-2	19 (63.3)	11 (36.7)	30 (100)
34a-1	30 (100)	0 (0)	30 (100)
21-2	07 (23.3)	23 (76.7)	30 (100)
106b-1	12 (40.0)	18 (60.0)	30 (100)
122a-1	23 (76.7)	07 (23.3)	30 (100)

**Table-III:** Comparison of miRNAs relative fold change expression (Mean ± SE (p) between exposed and non-exposed individuals.

miRNAs	Non-exposed	Exposed with expression ≤ 10	Exposed with expression > 10
Let-7a-2	2.44 ± 0.26	2.22 ± 0.65^#^	193.12 ± 71.55^***^
34a-1	1.50 ± 0.16	2.86 ± 0.53^**^	--
21-2	2.32 ± 0.34	3.02 ± 1.31^#^	182.34 ± 39.04^***^
106b-1	1.61 ± 0.15	3.01 ± 0.77^**^	20.44 ± 3.60^***^
122a-1	2.46 ± 0.43	3.23 ± 0.68^#^	20.43 ± 5.0^***^

Relative fold change expressions normalized with healthy control population shown in the columns. Mean and standard errors are listed with p values included in parentheses verses non-exposed; #p<0.1; *p < .05; **p < .001***p < .0001.

No significant association was found between age groups and working durations with expression levels ≤ 10 and > 10 for all five miRNAs (*p* > .05). However, out of 30 exposed individuals miR-21-2 was found with high expression levels (> 10-fold) in 46.7% individuals in age group (with > 40 years and 43.3% in working duration with > 15 years and respectively (Table-IV).

**Table-IV:** Association of age and working duration with expression levels of exposed individuals.

Expression levels of miRNAs		Age Group (Years)		*p*	Working Duration (Years)		*p*
	
		≤ 40	> 40		≤ 15	> 15	
let-7a-2	≤ 10	6 (20.0)	13 (43.3)	0.789	8 (26.7)	11 (36.7)	0.858
	> 10	4 (13.3)	7 (23.3)		5 (16.7)	6 (20.0)	
miR-34a-1	≤ 10	11 (36.7)	19 (63.3)	NA	13 (43.3)	17 (57.7)	NA
	> 10	0 (0)	0 (0)		0 (0)	0 (0)	
miR-21-2	≤ 10	1 (3.3)	6 (20.0)	0.222	3 (10)	4 (13.3)	0.977
	> 10	9 (30.0)	14 (46.7)		10 (33.3)	13 (43.3)	
miR-106b-1	≤ 10	3 (10.0)	9 (30.0)	0.429	4 (13.3)	8 (26.7)	0.367
	> 10	7 (23.3)	11 (36.7)		9 (30)	9 (30.0)	
miR-122a-1	≤ 10	7 (23.3)	16 (53.3)	0.542	9 (30)	14 (46.7)	0.400
	> 10	3 (10)	4 (13.3)		4 (13.3)	3 (10.0)	

## DISCUSSION

Previously TNT related hepatotoxicity and hematological diseases have been reported in military and munitions during both world wars.^11^ Trinitrotoluene is regarded as a carcinogenic agent in human and its exposure is associated with various cancers including leukemia^12^ and hepatocellular carcinoma.^13^ Although the role of miRNAs profiling in cancer and other diseases has been proposed,^14^ but the early monitoring to detect altered expressions of miRNAs in TNT exposed workers is unexplained/unknown. In the present study, the circulating miRNAs were measured in plasma of factory workers who were directly exposed to TNT to assess the extent of expression levels. Two miRNAs comprising let-7a-2 and miR-21-2 showed the considerable high expression levels in ~ 36% and 76% of exposed individuals respectively. Elevated plasma let-7a levels are considered as screening and prognostic indicators in hepatocellular carcinoma,^15^ and acute myeloid leukemia;^16^ the two cancers associated with TNT exposure. In contrast, some studies have also found decreased serum let-7a levels in multiple myeloma^17^ and bladder cancer.^18^ These studies, seemingly with some contradictory findings, point out the need for further investigations. In concordance with previous studies miR-21 has been found to be overexpressed in various cancers like acute myeloid leukemia and multiple myeloma.^19^ Studies also have shown that miR-21 may involve modulating PTEN tumor suppressor gene that leads to hepatocellular carcinoma.^20^ Thus, the dysregulation of these miRNAs may be more likely to be the indicators of future carcinomas in individuals exposed to TNT.

The present study shows the slightly increased up-regulation of miR-34a compared to rest of miRNA markers in TNT exposed individuals. MicroRNA-34a regulates its p53 target gene involved in tumor suppression in response of DNA damage which may indicate that the increased levels of miR-34 could function as a tumor suppressive miRNA with a protective effect.^21^ However, downregulation of miR-34a may lead to human liver carcinoma^22^ and acute myeloid leukemia.^23^

Circulating plasma levels of miR-106b and miR122a have also found significantly increases in the present study in TNT exposed individuals compared to healthy controls. In a study by Xuanjun Z et all., the urinary levels of miR-106b were significantly higher in bladder cancer patients than controls (P < 0.001).^24^ Studies have also shown that miR-122 is exclusively up-regulated in response of toxic liver injury,^25^ supporting the finding of increases of miR-122a may possibly in response of TNT exposure.

Our study suggests that these non-invasive circulating miRNAs could possibly be up regulated in response to direct exposure to TNT and might be involved in development of cancerous or other diseases based on miRNAs evaluation.

### Limitations of the study

A limitation of this study is lack of data on development of various cancers among local ordinance factory workers exposed to TNT, because of which it was not possible to ascertain prior association of TNT exposure with miRNAs. Another limitation of this study was less number of exposed participants. However, a larger scaled investigation *in-vivo* and *in-vitro* will be necessary to find out the association of dysregulation of these miRNAs using TNT as a toxic and carcinogenic agent.

## CONCLUSION

The study shows upregulation of all five miRNAs in TNT exposed subjects with no significant association of expression levels with age and working duration.

### Recommendations of the study:

1. Prospective studies using large sample may be caried out to establish definitive association of TNT exposure with dysregulation of miRNAs.
2. Follow up of TNT exposed individuals showing dysregulation of miRNAs to find out overt development of cancer.

### Authors Contribution:

**MA:** conceived, designed, and did biochemical analysis, statistical analysis and is responsibility for integrity of study.

**AKN:** Reviewed and final approval of manuscript.

**MA:** Molecular analysis, interpretation & writing of results and editing.

**AS:** Critical revision and editing of manuscript.

## References

[1] Shinkai Y, Li S, Kikuchi T, Shimojo N, Kumagai Y (2015). Health survey of workers in a 2,4,6-trinitrotoluene explosives factory in Fuxin, China. Fundam Toxicol Sci.

[2] Naderi M, Ghanei M, Shohrati M, Saburi A, Babaei M, Najafian B (2013). Systemic complications of trinitrotoluene in exposed workers. Cutan Ocul Toxicol.

[3] Kolb G, Becker N, Scheller S, Zugmaier G, Pralle H, Wahrendorf J (1993). Increased risk of acute myelogenous leukemia (AML) and chronic myelogenous leukemia (CML) in a county of Hesse, Germany. Soz Praventivmed.

[4] Katayama Y, Maeda M, Miyaguchi K, Nemoto S, Yasen M, Tanaka S (2012). Identification of pathogenesis-related microRNAs in hepatocellular carcinoma by expression profiling. Oncol Lett.

[5] Xie KL, Zang YG, Liu J, Zeng Y, Wu H (2014). MicroRNAs associated with HBV infection and HBV-related HCC. Theranostics.

[6] Yoo BK, Santhekadur PK, Gredler R, Chen D, Emdad L, Bhutia S (2011). Increased RNA-induced silencing complex (RISC) activity contributes to hepatocellular carcinoma. Hepatol.

[7] Meng F, Henson R, Wehbe-Janek H, Ghoshal K, Jacob ST, Patel T (2007). MicroRNA-21 regulates expression of the PTEN tumor suppressor gene in human hepatocellular gene. Gastroenterol.

[8] Johnson SM, Grosshans H, Shingara J, Byrom M, Jarvis R, Cheng A (2005). RAS is regulated by the let-7 microRNA family. Cell.

[9] Yamakuchi M, Ferlito M, Lowenstein CJ (2008). miR-34a repression of SIRT1 regulates apoptosis. Proc Natl Acad Sci USA.

[10] Moshiri F, Salvi A, Gramantieri L, Sangiovanni A, Guerriero P, De Petro G (2018). Circulating miR-106b-3p, miR-101-3p and miR-1246 as diagnostic biomarkers of hepatocellular carcinoma. Oncotarget.

[11] Boyer TD, Manns MP, Sanyal AJ Zakim and Boyer's Hepatology:A Textbook of Liver Disease. (Seventh Edition), Edit. Arun Sanyal Norah Terrault. Elsevier.

[12] Kilian PH, Skrzypek S, Becker N, Havemann K (2001). Exposure to armament wastes and leukemia:A case-control study within a cluster of AML and CML in Germany. Leuk Res.

[13] Sabbioni G, Sepai O, Norppa H, Yan H, Hirvonen A, Zheng Y (2007). Comparison of biomarkers in workers exposed to 2,4,6-trinitrotoluene, Biomarkers.

[14] Yang W, Lee DY, Ben-David Y (2011). The roles of microRNAs in tumorigenesis and angiogenesis. Int J Physiol Pathophysiol Pharmacol.

[15] Shi W, Zhang Z, Yang B, Guo H, Jing L, Liu T (2017). Overexpression of microRNA let-7 correlates with disease progression and poor prognosis in hepatocellular carcinoma. Medicine (Baltimore).

[16] Li Y, Lin J, Yang J, Qian J, Qian W, Yao DM (2013). Overexpressed let-7a-3 is associated with poor outcome in acute myeloid leukemia. Leuk Res.

[17] Manier S, Powers JT, Sacco A, Glavey SV, Huynh D, Reagan MR (2017). The LIN28B/let-7 axis is a novel therapeutic pathway in multiple myeloma. Leukemia.

[18] Qin MM, Chai X, Huang HB, Feng G, Li XN, Zhang J (2019). Let-7i inhibits proliferation and migration of bladder cancer cells by targeting HMGA1. BMC Urol.

[19] Handa H, Murakami Y, Ishihara R, Kimura-Masuda K, Masuda Y (2019). The role and function of microRNA in the pathogenesis of multiple myeloma. Cancers (Basel).

[20] Meng F, Henson R, Wehbe-Janek H, Ghoshal K, Jacob ST, Patel T (2007). MicroRNA-21 regulates expression of the PTEN tumor suppressor gene in human hepatocellular cancer. Gastroenterol.

[21] Zhou J, Zhou W, Kong F, Xiao X, Kuang H, Zhu Y (2017). MicroRNA 34a overexpression inhibits cell migration and invasion via regulating SIRT1 in hepatocellular carcinoma. Oncol Lett.

[22] Zhang HF, Wang YC, Han YD (2018). MicroRNA 34a inhibits liver cancer cell growth by reprogramming glucose metabolism. Mol Med Rep.

[23] Huang Y, Zou Y, Lin L, Ma X, Chen H (2018). Identification of serum miR-34a as a potential biomarker in acute myeloid leukemia. Cancer Biomark.

[24] Zhou X, Zhang X, Yang Y, Li Z, Du L, Dong Z (2014). Urinary cell-free microRNA-106b as a novel biomarker for detection of bladder cancer. Med Oncol.

[25] Wang K, Zhang S, Marzolf B, Troisch P, Brightman A, Hu Z (2009). Circulating microRNAs, potential biomarkers for drug-induced liver injury. Proc Natl Acad Sci USA.

